# Eye Tracking for Deep Learning Segmentation Using Convolutional Neural Networks

**DOI:** 10.1007/s10278-019-00220-4

**Published:** 2019-05-01

**Authors:** J. N. Stember, H. Celik, E. Krupinski, P. D. Chang, S. Mutasa, B. J. Wood, A. Lignelli, G. Moonis, L. H. Schwartz, S. Jambawalikar, U. Bagci

**Affiliations:** 1 Department of Radiology, Columbia University Medical Center - NYPH, New York, NY 10032 USA; 2 The National Institutes of Health, Clinical Center, Bethesda, MD 20892 USA; 3 Department of Radiology & Imaging Sciences, Emory University, Atlanta, GA 30322 USA; 4 Department of Radiology, University of California, Irvine, CA 92697 USA; 5 Center for Research in Computer Vision, University of Central Florida, 4328 Scorpius St. HEC 221, Orlando, FL 32816 USA

**Keywords:** Deep learning, Artificial intelligence, Segmentation, Meningioma, Eye tracking

## Abstract

Deep learning with convolutional neural networks (CNNs) has experienced tremendous growth in multiple healthcare applications and has been shown to have high accuracy in semantic segmentation of medical (e.g., radiology and pathology) images. However, a key barrier in the required training of CNNs is obtaining large-scale and precisely annotated imaging data. We sought to address the lack of annotated data with eye tracking technology. As a proof of principle, our hypothesis was that segmentation masks generated with the help of eye tracking (ET) would be very similar to those rendered by hand annotation (HA). Additionally, our goal was to show that a CNN trained on ET masks would be equivalent to one trained on HA masks, the latter being the current standard approach. Step 1: Screen captures of 19 publicly available radiologic images of assorted structures within various modalities were analyzed. ET and HA masks for all regions of interest (ROIs) were generated from these image datasets. Step 2: Utilizing a similar approach, ET and HA masks for 356 publicly available T1-weighted postcontrast meningioma images were generated. Three hundred six of these image + mask pairs were used to train a CNN with U-net-based architecture. The remaining 50 images were used as the independent test set. Step 1: ET and HA masks for the nonneurological images had an average Dice similarity coefficient (DSC) of 0.86 between each other. Step 2: Meningioma ET and HA masks had an average DSC of 0.85 between each other. After separate training using both approaches, the ET approach performed virtually identically to HA on the test set of 50 images. The former had an area under the curve (AUC) of 0.88, while the latter had AUC of 0.87. ET and HA predictions had trimmed mean DSCs compared to the original HA maps of 0.73 and 0.74, respectively. These trimmed DSCs between ET and HA were found to be statistically equivalent with a *p* value of 0.015. We have demonstrated that ET can create segmentation masks suitable for deep learning semantic segmentation. Future work will integrate ET to produce masks in a faster, more natural manner that distracts less from typical radiology clinical workflow.

## Introduction

Eye tracking (ET) has been used extensively for research in marketing, psychology, and medical image interpretation [1, 2]. It has also been used in medical imaging research to elucidate differences in how expert and novice radiologists interpret images [3–6]. For example, decades of work have used ET to predict radiologist diagnosis and detect the search path of various image modalities, including chest imaging [1, 2, 7], mammography [3, 8], CT colonography [9], dental radiographs [10], and musculoskeletal radiographs [11], based on radiologist gaze points within the images [12]. Other efforts have gone into integration of ET into standard clinical workflow [13]. Additional research has employed ET to study radiologist response to interruptions [14, 15] and fatigue [16, 17].

More recently, Khosravan et al. employed ET in an actual clinical practice setting to improve the detection, diagnosis, and quantification (segmentation) of lung nodules and prostate lesions [18, 19]. Segmentation is widely recognized as an important task in image quantification [20–22]. It has become an ad hoc component of most computer-assisted diagnosis (CAD) tools. The recent exponential growth of artificial intelligence (AI), specifically deep learning (DL) using convolutional neural networks (CNNs) has enabled the segmentation of anatomic structures previously difficult to segment with older methods of thresholding, region/boundary-based approaches, or hybrid techniques such as combining models and graph-search algorithms [20–22]. As such, AI holds great promise as a method to segment structures of interest (SOIs, e.g., organs or tumors) automatically, either in clinical practice or for research purposes. AI has shown promise for assisting in worklist triage [23], and automated segmentation will likely be an important component of this functionality.

A key limitation to the advancement of medical DL segmentation is obtaining abundant quantities of accurately curated imaging data for effective DL training. Specifically, what is needed to build successful CNNs is a large number and variety of images, with corresponding expert-annotated masks that specify per-pixel locations of SOIs. Typically, the task of annotation is done manually with mouse clicks, tracing out the borders of the SOI for each image and each image slice to be used in training the network. This approach has two drawbacks: (1) it can be very time-consuming, especially as larger, more complex datasets are needed; (2) it generally occurs outside of routine clinical care during dedicated research time. As such, these tasks are often done by nonradiologist researchers who, given less clinical exposure to those types of images and structures, may not recognize as well the correct SOIs or their proper borders, reducing annotation accuracy.

Therefore, a method to annotate medical images during routine clinical work could provide far greater efficiencies. Such annotation would be performed by expert clinicians, radiologists, or pathologists, often subspecialized in the particular subset of images they interpret clinically [24]. Additionally, this work could reduce outside time commitment, since it would be done as part of the normal clinical work already being performed. With tens of millions of magnetic resonance images (MRI) and CT scans being performed annually in the USA alone, harvesting even a small portion of this clinical volume for deep learning would represent a tremendous leap forward.

We propose ET as a technology to allow routine clinical interpretation of images to generate annotated data for DL algorithm training. As radiologists’ and pathologists’ clinical workflow is centered around visually examining images, ET can capture when they are gazing at SOIs. In the case of radiologists, classification information is also available for recording during clinical work because they typically dictate labels or descriptions of SOIs into a voice recognition system while gazing at the structures.

In order to evaluate the potential of such a technique, we sought to assess if ET can generate image masks equivalent to those rendered by the conventional hand annotation (HA) for DL segmentation.

In step 1 of the present study, we hypothesized that masks produced by ET would be similar to those obtained from HA for a wide variety of image modality and region of interest (ROI) types.

In step 2, we hypothesized that, for a set of meningioma images, ET image masks would not only be similar to HA masks but also would generate equally accurate DL segmentation predictions compared to those from HA.

## Methods

### Image Acquisition

#### Step 1

We obtained 19 publicly available nonneurological radiologic images from Google Images and PubMed Images [25, 26]. The goal here was not to use masks for DL, but simply to demonstrate that effective mask generation from ET is possible with a diverse array of SOIs for a wide variety of imaging modalities.

The set of images used in this study (for both steps 1 and 2) is listed in Table 1. The much larger number of meningioma images was chosen so as to provide enough data for DL, and we included normal brain images to increase CNN specificity. We generated segmentation masks without DL for a few examples of various types of nonmeningioma images in order to illustrate that this is indeed a very broad and general approach. It can in theory segment any type of object in any type of medical image.

**Table 1:** Enumeration of the number and types of imaging modalities and structures of interest for which masks were generated both by eye tracking and by hand annotation. In the case of meningiomas, the masks were subsequently used to train and test a segmentation CNN

Image type	Number of images
Meningiomas on postcontrast T1WI	356
Normal brains on postcontrast T1WI	69
Livers on CT	5
Breast masses on mammography	4
Kidneys on ultrasound	5
Heart on PET MIP	5

#### Step 2

We obtained 356 publicly available postcontrast T1-weighted MRI of meningiomas, 23 of them through publicly available images on Google Images, and the other 333 from peer-reviewed publications via PubMed Images [25, 26]. All images were screen captured as PNG files on a Macbook Air (Apple, Cupertino, CA). Additionally, 69 T1 postcontrast images of normal brains were obtained as screen captures from a publicly available image database [27]. Most images had been obtained in the axial plane, though approximately 25% were in sagittal or coronal planes. All images were loaded into a PowerPoint (Microsoft, Redmond, WA) document to allow for rapid viewing and ET annotation, although this could have also be done with a DICOM viewer.

### Eye Tracking

#### Steps 1 and 2

The Fovio™ Eye Tracker remote eye-tracker system (Seeing Machines Inc., Canberra, Australia) was used in this study. Gaze data were collected using the associated EyeWorks™ Suite (v.3.12) at the rate of 60 Hz on a DELL Precision T3600 (Windows 7, Intel Xeon CPU E5−1603@2.80 GHz with 128 GB of RAM). Images were presented on a 30″ EIZO LCD monitor. Displayed images were fit to as large a rectangular space as feasible on the display monitor. Initial calibration was confirmed by way of a nine-point calibration procedure in EyeWorks™ Record. Calibration was also reconfirmed manually by the operator after initial calibration was done and before the image viewing started; confirmation required that the user’s gaze matched the location of an alternately moving and stopping display point on the monitor to within a preprogrammed threshold value.

The Fovio remote system was located 2 cm beneath the bottom of the viewing screen and at a 26° angle with respect to the monitor. Following calibration, the user completed the task of visually tracing gaze around the meningioma or other SOI borders. A single radiologist trained in neurologic image interpretation (JNS) viewed the images. Zoom/pan and window level adjustments were not permitted.

**Fig. 1:**
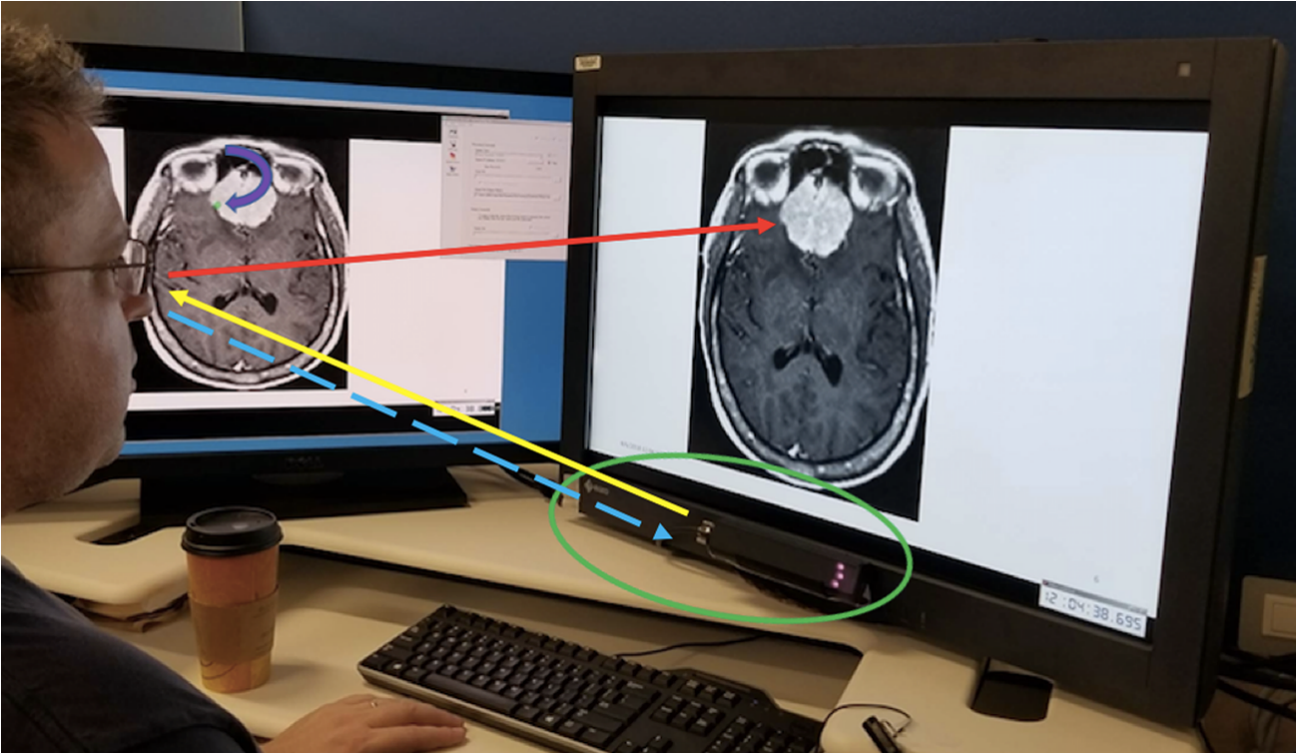
Eye-tracking setup. User gaze direction is shown by the red arrow. Eye-tracking hardware (in green oval) sends near-infrared light (yellow arrow) to the user’s eyes, which are reflected back as the corneal reflection (dashed blue arrow). The calibration software is able to calculate from the latter the user’s gaze position (curved purple arrow pointing toward green dot on the monitor to the user’s left) in real time

A screen captured wmv format video file with continuous recording of time points was generated. The task of the radiologist was to explicitly move his eyes around every meningioma and other SOI surface/outer contour. The video displayed user gaze position as a function of time overlaid on the images. This was essentially a video of the user’s gaze during the entire eye-tracking process. The total time spent viewing all 356 meningioma borders plus the 19 other SOI borders, which became the video file running time, was 2280.3 s, or approximately 38 min. A schematic of the eye-tracking setup is shown in Fig. 1.

The software calculates fixations (where the eyes land with foveal vision). Fixation locations and dwell times are automatically correlated with x,y image locations. The system’s assessment of x,y locations was based on an internal reference system within the monitor, not specific to PowerPoint. Thus, we were able to identify which fixations were associated with the meningiomas and other SOIs for each image. Fixations associated with the user looking elsewhere (i.e., nonmeningiomas) were excluded. From these data, an image trace was generated for each image using the EyeWorks Analyze software. This consisted of pixel values equal to one at the locations of gaze and zero elsewhere.

Detailing further, non-SOI fixation points were excluded as follows: the video file of the real-time gaze fixation points on the set of images was reviewed after the entire ET exercise was performed. This video also contained a recording of the timer that accompanied the gazes. Hence, the video included within it the set of all time points ${\left\{{t}_j\right\}}_{j=1}^{N_t}$, where *N*_*t*_ is the total number of time points that transpired during image examination with ET. For our ET session and corresponding video, which lasted roughly 2300 s, with 100-ms increments, *N*_*t*_ ≈ 2.3 × 10^4^.

We shall denote the set of all meningiomas as ${\left\{{M}_i\right\}}_{i=1}^{N_M}$, where *N*_*M*_ = 356 is the total number of meningiomas examined. For a given meningioma *M*_*i*_, typically the first second or so of gaze fixation would not be on the lesion. However, as soon as the SOI was detected by the user in his peripheral vision, he switched gaze to focus on the meningioma borders. During video review, the time at which the user’s gaze first fell on the meningioma borders was noted and recorded in an Excel spread sheet as the start time for that meningioma, which we denote as ${t}_{\mathrm{start}}^{\left({M}_i\right)}$. We denote *x*,*y* position of the gaze point at ${t}_{\mathrm{start}}^{\left({M}_i\right)}$ by $\left({x}_{t_{\mathrm{start}}^{\left({M}_i\right)}}\kern0.5em {y}_{t_{\mathrm{start}}^{\left({M}_i\right)}}\right)$. When the user was done performing a counterclockwise trace around the meningioma with his eyes, he clicked on the keyboard’s down arrow to bring up the next meningioma image. This having been recorded in the video, upon subsequent video review, a corresponding time was displayed in the video. This time showed when the user last looked at the meningioma border; this was recorded as the stop time, ${t}_{\mathrm{stop}}^{\left({M}_i\right)}$, for meningioma *M*_*i*_, with corresponding gaze point position $\left({x}_{t_{\mathrm{stop}}^{\left({M}_i\right)}}\kern0.5em {y}_{t_{\mathrm{stop}}^{\left({M}_i\right)}}\right)$.

Hence, for meningioma *M*_*i*_, the border trace is extracted as the set of *x*,*y* points:

$$ \left({\overrightarrow{x}}_{M_i},{\overrightarrow{y}}_{M_i}\right)\equiv \left(\begin{array}{cc}{x}_{t_{\mathrm{start}}^{\left({M}_i\right)}}& {y}_{t_{\mathrm{start}}^{\left({M}_i\right)}}\\ {}{x}_{t_{\mathrm{start}}^{\left({M}_i\right)}+1}& {y}_{t_{\mathrm{start}}^{\left({M}_i\right)}+1}\\ {}\begin{array}{c}\vdots \\ {}{x}_{t_{\mathrm{stop}}^{\left({M}_i\right)}}\end{array}& \begin{array}{c}\vdots \\ {}{y}_{t_{\mathrm{stop}}^{\left({M}_i\right)}}\end{array}\end{array}\right) $$

### Mask Generation, Training, and Testing

#### Steps 1 and 2

Image contour traces were imported into Matlab. Masks were generated as the convex hull of the traces. At a later date, hand annotations of every meningioma and other SOI image were obtained by the same radiologist (JNS) via the roipoly function in Matlab. For the normal postcontrast images without meningioma, masks were simply the zero matrix. All images (anatomical and their corresponding masks) were resized in Matlab to be 256 × 256 pixels.

#### Step 2

The meningioma postcontrast images along with the ET and HA masks were imported into Python as *mat files. The images were normalized to have zero mean and unit standard deviation, in order to reduce network susceptibility to covariate shift. At this point, 306 of the 356 images were assigned randomly for training and validation, while the other 50 images were reserved as a testing set. Of the 306 training/validation images, 80% were randomly selected for training, while the other 20% for were used for validation.

Training was performed using Python 3.7.0 and the packages Tensorflow and Keras on a workstation with three 1080 NVIDIA Ti GPUs (11 GB each) with two 18-core 2.10 Gigahertz Intel Xeon E5-2695 v4 CPUs and 256 GB system RAM.

The meningioma images were used as input to a 20-layer neural network based on the U-net architecture [28] which has been shown to be effective for medical image segmentation [29–31]. Initial filter parameter weights were randomly selected with a mean of zero according to the default Keras Glorot initialization. All convolutions were performed with 3 × 3 filters. We used negative Dice coefficient as the loss function for training. A steepest gradient descent approach based on the Adam optimization algorithm with step length 1 × 10^−5^ was used. The model was trained for 600 epochs, after which improvement in accuracy of the prediction and loss values leveled off. Training time was approximately 6 h using both ET and HA masks.

The 50 testing set meningioma images were run through the trained CNN. Four examples of testing set meningioma images with superimposed CNN mask predictions are provided in Fig. 4. No size threshold was applied to those predictions.

Receiver operating characteristic (ROC) area under the curve (AUC) was calculated by varying size thresholds for predicted pixel groups or “clusters” in a manner very similar to that used in previous work segmenting cerebral aneurysms [32]. We used the matplotlib Python plotting library to generate and display the ROC curves. It is worth noting that the ROC curves both for HA- and ET-based predictions were generated by comparing predicted masks to HA mask annotation as the gold standard. Using the HA mask (the one initially traced around the meningioma, not the one predicted by the CNN trained on HA masks) as the truth in this manner was felt to constitute a stricter criterion to test the ET method.

In order to show equivalence of ET and HA masks, we used the two one-sided test (TOST) method [33, 34]. TOST is a complement to the *t* test method, which is used to determine whether there is a statistically significant difference between the means of two datasets. We sought to show the opposite: that our two datasets, namely the set of ET mask predictions and the set of HA mask predictions, were statistically equivalent. Not being able to demonstrate statistically significant difference on a *t* test does *not* show equivalence, hence the need for an equivalence test like TOST.

Equivalence is shown when the difference Δ between two sets falls between a predetermined range −Δ_*L*_ < Δ < Δ_*U*_, where Δ_*L*_ and Δ_*U*_ are the lower and upper equivalence bounds, respectively. These bounds are prespecified to satisfy the smallest effect size of interest (SESOI). The SESOI is essentially a threshold for how close the values from two datasets should be in order to be considered equivalent [33, 34].

## Results

### Step 1

**Fig. 2:**
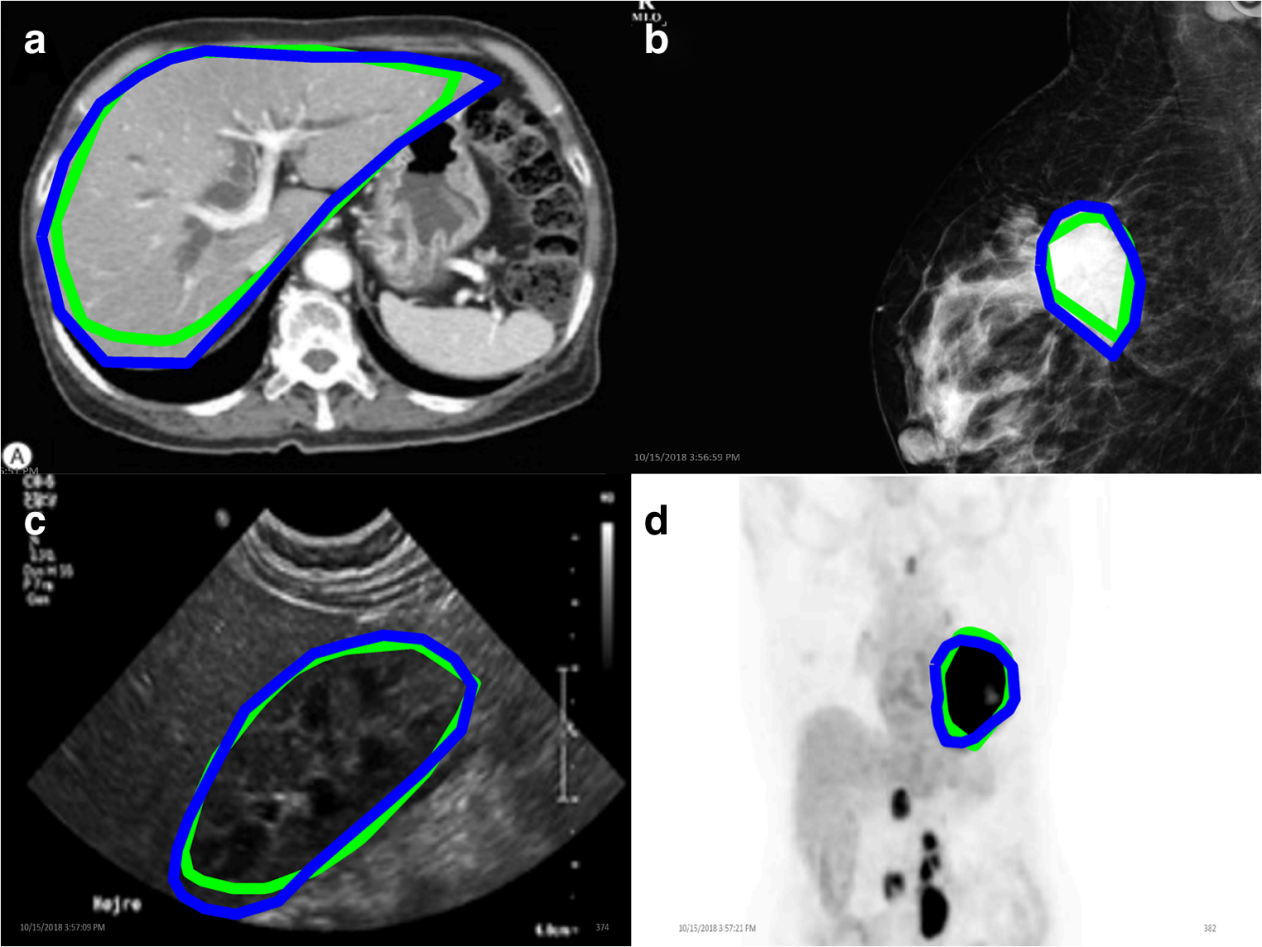
Nonneurological image mask borders of various structures of interest. Eye-tracking mask contours are in green, hand annotation in blue

For the 19 nonmeningioma, nonbrain MRI images, the average Dice coefficient between HA and ET was 0.86. Four examples of the surface boundaries of the masks generated by the two methods are shown in Fig. 2.

### Step 2

**Fig. 3:**
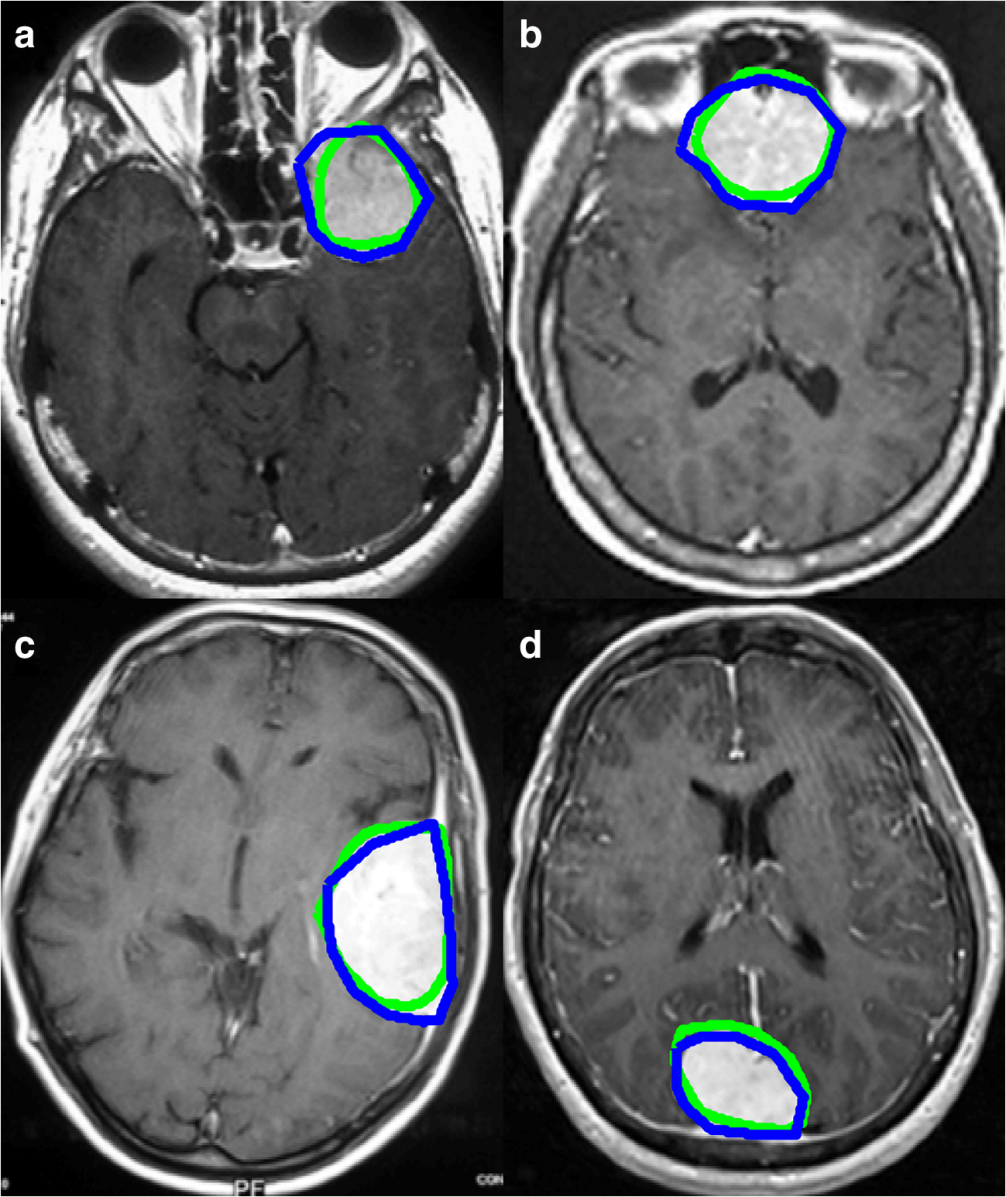
Four examples of meningioma mask border comparisons. Eye-tracking (green) and hand annotation (blue) mask contours are overlaid with the corresponding postcontrast T1-weighted images containing meningiomas

For the 356 meningioma-positive images with nonzero masks generated both by HA and ET, the mean Dice coefficient for overlap between the two mask types was 0.85. Four examples of the outer border of the masks generated by HA and ET are displayed in Fig. 3.

The ROC curves both for HA and ET methods are shown in Fig. 5. Both methods give AUC values of close to 0.9 (0.87 for HA, 0.88 for ET), and the curves are essentially the same for the two methods. Additionally, the trimmed mean DSC for CNN-predicted mask versus HA generated mask was 0.74 using HA masks as model input, while for using ET as model inputs, it was virtually identical at 0.73. The DSCs for HA and ET were statistically equivalent by the TOST method [33, 34] with a *p* value of 0.015, using 0.1 as the SESOI.

## Discussion

### Discussion of Results

As can be seen in Fig. 2, the mask surface boundaries from HA and ET match fairly closely, which is compatible with the high mean Dice similarity score between them. However, it should be noted that in Fig. 2a, the ET trace is noted to “cut corners” around the liver compared to HA. This likely resulted from the fact that ET annotation of nonmeningioma images was performed at the end of one long session after ET meningioma annotation for all 356 meningioma images. Hence, there was probably a fatigue factor leading to the short cut seen in Fig. 2a. Hence, this nicely illustrates the fatigue-related limitation of our particular first iteration of the approach involving consciously tracing one’s gaze around lesion borders. Figure 3c shows one mildly “cut corner” around the meningioma, indicating that not only fatigue but possibly a neurobehavioral/neurocognitive tendency of the user was to take this minor shortcut while moving the eyes but not when tracing borders by hand. Ultimately, small differences such as these did not engender a significant difference between the overlap of the masks themselves nor the performance of the resulting ET CNN versus the HA CNN, as verified by mean Dice scores all 85% or greater.

**Fig. 4:**
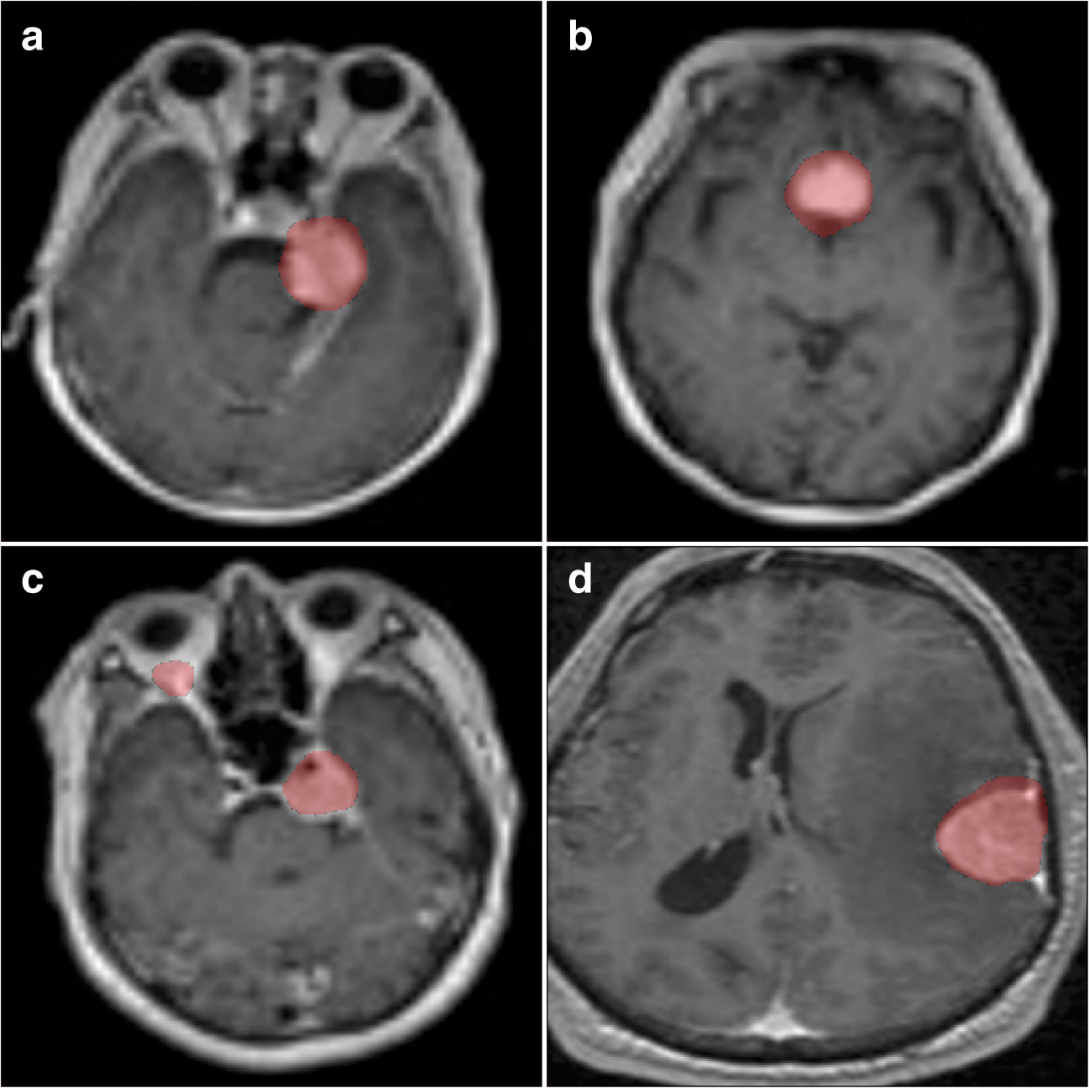
Four example predictions from the CNN trained on eye-tracking-generated images. Predicted meningioma pixels are in red. Significant overlap between the predicted masks and the enhancing lesions is evident. Note the false positive pixels in Fig. 4c in the right orbital fat. The algorithm is mistaking the high signal intensity of fat for enhancing meningioma

Figure 4 shows significant overlap between the CNN-predicted masks and the enhancing lesions. Note the false positive pixels in Fig. 4c in the right orbital fat. Here, the algorithm is mistaking the high signal intensity of fat for enhancing meningioma. However, in our experience, the more images the CNN was trained on that had orbits (without a mask over them), the better the trained network was at discerning that these were not meningioma, and the fewer of these errant predictions were seen. Also, postprocessing with size thresholds would remove smaller errant predictions such as this, as implemented in calculating the ROC curves.

**Fig. 5:**
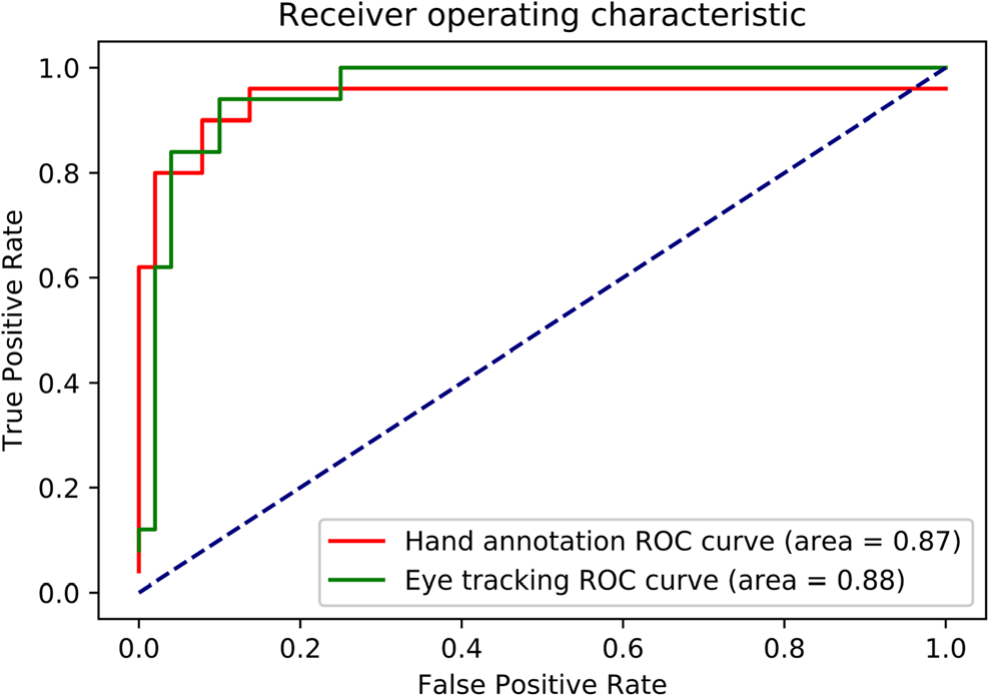
ROC curve. Eye-tracking curve is in green, while hand annotation curve is in red

Most importantly, Fig. 5 shows the overall performance of both HA- and ET-generated CNNs to be essentially identical, which is further verified by their near-equivalent mean Dice scores, both over 85% and statistically equivalent.

Overall, we have thus demonstrated that ET can be an effective approach for generating segmentation masks for DL.

### Limitations and Future Directions

There are several limitations in this study. Only one radiologist was tested for this eye-tracking study, and this is the same radiologist who performed the manual draws. The results, therefore, may not be entirely applicable to all radiologists. Additionally, many of the lesions selected for contouring were relatively large, and thus, the eye-tracking results were likely more accurate than if only smaller lesion targets had been used.

We chose the method of screen capturing publicly available images as PNG files for practicality, expediency and as a safeguard to avoid compromising protected health information. However, the images obtained were of significantly lower quality than DICOM files captured in full spatial resolution and number of grayscale levels from a local Picture Archiving and Communication System (PACS) database. Using lower image quality for training and/or testing CNNs has been shown to yield worse results than for higher quality images [35, 36]. As such, it is notable that our CNNs achieved fairly high AUC values with these comparatively low-quality images.

An important ultimate goal would be to integrate this technology into routine clinical practice, so that potentially millions of mask annotations could be harvested from clinical work. The approach presented here is a proof-of-principle and is far from perfected. Tracing out lesion borders visually is tedious, tiring, and divorced from how radiologists view structures of interest during image interpretation. Hence, a more “ecologically valid” workflow is needed. A combination of eye-tracking data, thresholding, and iterative deep learning may provide the eventual best route. Future work will thusly endeavor to develop such hybrid approaches.
